# Design and Characterization of Novel Polymeric Hydrogels with Protein Carriers for Biomedical Use

**DOI:** 10.3390/ijms26010258

**Published:** 2024-12-30

**Authors:** Magdalena Kędzierska, Magdalena Bańkosz, Katarzyna Sala, Claudia Garbowska, Oliwia Grzywacz, Wiktoria Wrzesińska, Aneta Liber-Kneć, Piotr Potemski, Bożena Tyliszczak

**Affiliations:** 1 Department of Chemotherapy, Medical University of Lodz, Copernicus Memorial Hospital of Lodz, 90-549 Lodz, Poland; magdalena.kedzierska@umed.lodz.pl (M.K.); piotr.potemski@umed.lodz.pl (P.P.); 2 Department of Materials Engineering, Faculty of Materials Engineering and Physics, CUT Doctoral School, Cracow University of Technology, 37 Jana Pawla II Av., 31-864 Krakow, Poland; 3 Department of Materials Engineering, Faculty of Materials Engineering and Physics, Cracow University of Technology, 37 Jana Pawla II Av., 31-864 Krakow, Poland; katarzyna.sala@student.pk.edu.pl (K.S.); claudia.mordaka@student.pk.edu.pl (C.G.); oliwia.grzywacz@student.pk.edu.pl (O.G.); wiktoria.wrzesinska@student.pk.edu.pl (W.W.); 4 Department of Applied Mechanics and Biomechanics, Faculty of Mechanical Engineering, Cracow University of Technology, 37 Jana Pawla II Av., 31-864 Krakow, Poland; aliber@pk.edu.pl

**Keywords:** DDS, hydrogel modification, photopolymerization, cisplatin

## Abstract

Hydrogels are three-dimensional polymeric matrices capable of absorbing significant amounts of water or biological fluids, making them promising candidates for biomedical applications such as drug delivery and wound healing. In this study, novel hydrogels were synthesized using a photopolymerization method and modified with cisplatin-loaded protein carriers, as well as natural extracts of nettle (*Urtica dioica*) and chamomile (*Matricaria chamomilla* L.). The basic components of the hydrogel were polyvinylpyrrolidone and polyvinyl alcohol, while polyethylene glycol diacrylate was used as a crosslinking agent and 2-methyl-2-hydroxypropiophenone as a photoinitiator. The hydrogels demonstrated high swelling capacities, with values up to 4.5 g/g in distilled water, and lower absorption in Ringer’s solution and simulated body fluid (SBF), influenced by ionic interactions. Wettability measurements indicated water contact angles between 51° and 59°, suggesting balanced hydrophilic properties conducive to biomedical applications. Surface roughness analyses revealed that roughness values decreased after incubation, with Ra values ranging from 6.73 µm before incubation to 5.94 µm after incubation for samples with the highest protein content. Incubation studies confirmed the stability of the hydrogel matrix, with no significant structural degradation observed over 20 days. However, hydrogels containing 2.0 mL of protein suspension exhibited structural damage and were excluded from further testing. The synthesized hydrogels show potential for application as carriers in localized drug delivery systems, offering a platform for future development in areas such as targeted therapy for skin cancer or other localized treatments.

## 1. Introduction

In recent years, hydrogel materials have attracted significant scientific attention due to their diverse applications across multiple scientific domains. These materials are three-dimensional polymer networks that can absorb large quantities of water or other biological fluids [1,2]. In the biomedical sector, hydrogels have extensive applications, including controlled drug release, bone repair, and tissue engineering. With current challenges in disease treatment, particularly in oncology, advancements in science and technology are pivotal in the development of more effective and personalized therapeutic strategies. Hydrogels are widely regarded as promising materials for anticancer drug delivery due to their ability to enable localized drug release directly at the tumor site. This localized delivery mechanism is proposed to reduce systemic toxicity and achieve high concentrations of the therapeutic agent at the specific location where it is most needed. Such systems are particularly relevant in the treatment of solid tumors, including skin cancers, where hydrogels can be applied topically or subcutaneously to directly target affected tissues. Additionally, hydrogels provide a controlled environment for drug release, which may help maintain therapeutic drug concentrations over an extended period. This property could be especially advantageous in managing slow-growing tumors or in reducing the likelihood of recurrence after surgical excision [3,4]. It is recognized that this approach holds significant potential for cancers such as melanoma, basal cell carcinoma, or squamous cell carcinoma. A promising research direction involves creating multicomponent polymer systems, especially protein-based carriers, to facilitate targeted anti-cancer drug delivery. R. Vildanova et al. developed biodegradable hydrogels using natural polyelectrolytes, chitosan, and pectin. Their findings revealed that hydrogels with varied rheological properties and swelling behaviors could be produced by adjusting the molecular weight of pectin [5]. In addition, it is worth noting that hydrogels possess a remarkable ability to absorb water, resulting in the formation of three-dimensional structures with regulated porosity [6,7,8]. This characteristic facilitates the straightforward incorporation of drugs within the hydrogel matrix and enables controlled release from the carrier. Furthermore, the water-absorbing property of hydrogels helps maintain an optimal hydration level at the site of drug application, which is particularly beneficial for drugs administered to the skin or mucous membranes [9]. Equally significant is their high biocompatibility, rendering them suitable for various clinical uses. Additionally, their structural flexibility allows them to be readily tailored for multiple therapeutic purposes, including as carriers for topical applications, transdermal drug delivery systems, or in tissue engineering treatments [10,11,12].

In turn, albumin, the primary plasma protein, offers high biocompatibility, a strong capacity for binding and transporting various molecules, and is readily modified chemically [13]. As a result, albumin has been utilized as a carrier for anticancer drugs, enhancing their dispersibility, stability, bioavailability, and precision in targeting drug delivery to cancerous cells [14]. The function of albumin as a drug carrier relies primarily on two core mechanisms: (1) its capacity to bind a variety of drug molecules and (2) its selective interactions with receptors located on the surfaces of cancer cells [15,16]. Due to its multiple binding sites, albumin can associate with both hydrophobic and hydrophilic drugs, forming stable drug-albumin complexes. Furthermore, albumin exhibits a preferential affinity for receptors, such as glycoprotein 60 (GP60), located in tumor vasculature, facilitating directed drug delivery to the intended site. The application of albumin as an anticancer drug carrier may offer several clinical advantages, such as enhanced drug stability and retention in plasma, decreased toxicity to healthy tissues, and improved bioavailability and therapeutic effectiveness [17,18]. Additionally, the possibility of chemically modifying albumin allows the integration of specific functional groups, which can enhance targeting specificity to cancer cells and regulate the drug release rate from the carrier [19]. Moreover, albumin can be delivered using hydrogels, as described in the studies by Tincu et al. and Diaz and Missirlis, which presented methods for binding albumin with hydrogels of various structures, further confirming the potential of this approach in biomedical applications [20,21]. Additionally, Patel et al. investigated the use of injectable thermosensitive hydrogels for the sustained release of albumin-based therapeutics, highlighting the potential of these systems for noninvasive subcutaneous delivery. These findings emphasize the need for further exploration and optimization of hydrogel formulations to improve the efficiency and precision of albumin-based therapeutic delivery systems [22].

This study centers on the integration of albumin carriers with hydrogel materials. It is assumed that the obtained materials could be utilized in the local treatment of cancers such as melanoma, basal cell carcinoma, or squamous cell carcinoma in the form of transdermal systems. In these systems, a cytostatic drug combined with an albumin-based carrier is encapsulated within a polymer matrix. This design aims to enable localized and prolonged drug release, ensuring effective therapeutic action at the diseased site while minimizing systemic toxicity. During synthesis, cisplatin was chosen as an anticancer drug. This drug is known for its high efficacy, but it has some limitations related to systemic toxicity [23,24]. Integrating protein carriers, such as albumin, with hydrogel structures leverages the distinct properties of both components to enhance drug stability and delivery efficiency. Albumin, a natural carrier for numerous bioactive molecules, enhances the bioavailability and stability of drugs, especially those with low water solubility. Furthermore, interactions between albumin and the hydrogel matrix can result in stable complexes that prevent premature drug release, enabling controlled drug delivery to target sites. This integration allows for targeted drug delivery to specific tissues or cells, enhancing therapeutic efficacy while reducing adverse effects. Moreover, both albumin and hydrogels can be chemically modified, allowing for the addition of specific functional groups to improve drug-binding specificity and regulate release rates from the carrier [25,26,27,28].

In this study, hydrogels modified with two plant extracts, nettle (*Urtica dioica*) and chamomile (*Matricaria chamomilla* L.), were synthesized. These materials were designed to function as protein carriers, incorporating albumin along with a cytostatic drug. Physicochemical analyses were conducted to assess the sorption capacity of these materials, which were also subjected to incubation tests in simulated body fluids. Additionally, the surface properties of the materials were examined. Findings indicated that all materials demonstrated high sorption capacities, with surfaces characterized by moderate roughness. The inclusion of plant extracts within the hydrogel matrices introduced an additional research dimension, presenting novel opportunities in the development of biomaterials for biomedical applications. The aim of this study was to develop and characterize hydrogel materials incorporating albumin-based carriers, considering their potential for various biomedical applications. With the inclusion of cytostatic drug carriers and natural extracts such as nettle and chamomile, these materials show promise for use in both anticancer therapy and supporting wound healing processes. This article presents preliminary research aimed at determining the potential of these materials for further applications.

## 2. Results and Discussion

### 2.1. Analysis of Sorption Capacity

**Figure 1:**
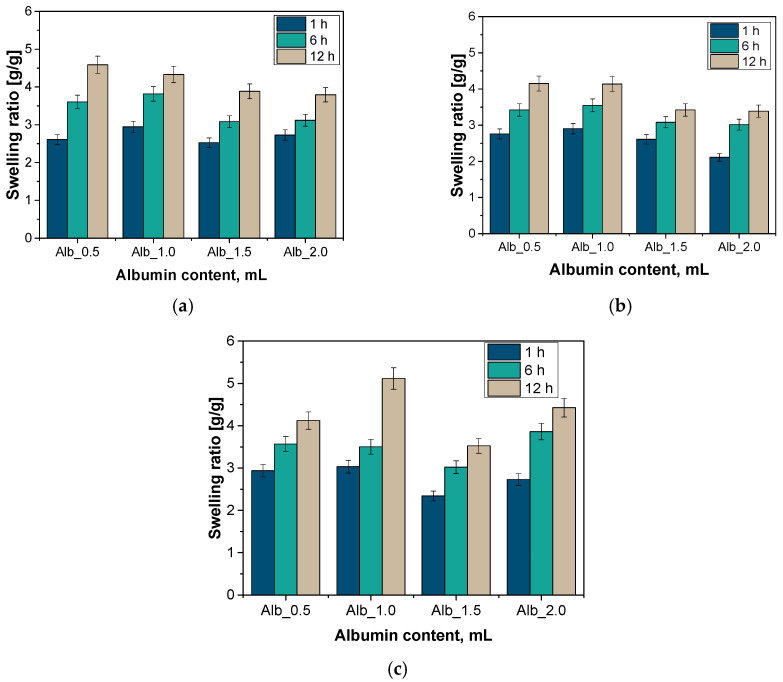
Results of sorption capacity analysis in SBF liquid (**a**) Ringer’s solution (**b**) and distilled water (**c**). (*n*—number of repetitions, *n* = 3).

Albumin was incorporated into the hydrogel matrix by forming cisplatin-loaded protein spheres through a precipitation process. These spheres were uniformly distributed within the hydrogel during polymerization. Next, to investigate and compare the sorption capacity of hydrogel materials, a swelling analysis was conducted. This study considered the impact of varying amounts of added protein sphere suspension as well as the inclusion or exclusion of specific plant extracts. The results of this analysis for SBF, Ringer’s solution, and distilled water are presented in the graphs below (Figure 1). Statistical analysis is presented in Table 1.

The analysis indicates that all synthesized hydrogel materials exhibit measurable swelling capacities. Notably, the swelling kinetics reveal a sharp increase in sorption capacity within the first hour, followed by a gradual stabilization of swelling coefficient values over time. During the initial hours, the sorption activity is especially pronounced, as the hydrogel material rapidly absorbs substantial amounts of the incubation fluid. This initial rapid absorption is linked to the abundant availability of sorption sites. Over time, however, the sorption rate diminishes, which can be attributed to various factors. Primarily, as the material approaches its maximum sorption capacity for a given fluid, a saturation process reduces the availability of open sorption sites. As these sites become occupied, the absorption rate declines. Furthermore, internal diffusion processes grow increasingly complex with higher liquid absorption, further influencing the slowing of sorption rates. The initial surge in sorption likely reflects the polymer matrix’s interaction with the liquid, while the subsequent reduction in sorption rate is associated with material saturation and the onset of internal diffusion, which limits the available space for absorbed liquid. Similar findings were reported by Díaz-Marín et al. in their study [29].

The analysis demonstrated that hydrogels exhibit a greater sorption capacity in distilled water compared to Ringer’s fluid and SBF solution. This difference can be attributed to the distinct chemical compositions of these environments. Both Ringer’s fluid and SBF solution contain additional salts and ions that may compete for sorption sites within the hydrogel structure, thereby limiting the hydrogel’s capacity to absorb water and other substances. Furthermore, the ions present in Ringer’s and SBF solutions can induce electrostatic interactions with the hydrogel, which further restricts fluid movement within the material. Distilled water, having a significantly lower ion concentration than Ringer’s fluid or SBF, reduces such competition for binding sites, leading to higher absorption levels in this medium. Similarly, Feng et al. observed that water sorption decreases as the ion concentration in the swelling medium increases [30].

The analysis of the hydrogel material’s sorption capacity in relation to the albumin carrier content reveals notable changes in sorption behavior as albumin levels increase. This effect is likely due to albumin’s role in altering the surface characteristics of the hydrogel, potentially influencing sorption by forming complexes with cytostatic drug molecules. With higher albumin content, the hydrogel’s surface available for sorption may be reduced due to the albumin molecules coating it, leading to a decrease in sorption capacity. Additionally, an elevated concentration of albumin carriers may enhance the binding of cytostatic drugs through interactions with albumin’s functional groups, further limiting the free sorption sites for the drug. Interestingly, the reduction in sorption capacity in hydrogels with higher albumin content can be advantageous for controlled drug delivery, as it may slow drug release rates and enhance drug stability. Studies by Zhang et al. and Noh et al. highlighted that variations in hydrogel swelling capacity could result from differences in microstructure. They investigated hydrogels based on methacrylate hyaluronic acid with the addition of acrylate bisphosphonate (Ac-BP) as a comonomer. This addition increased crosslinking density, subsequently reducing swelling capacity [31,32].

### 2.2. Results of the Incubation Study

**Figure 2:**
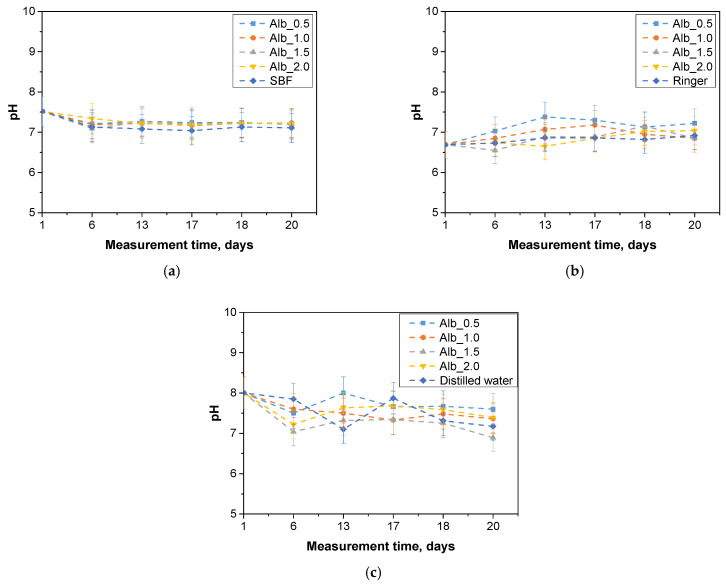
Results of incubation analysis in SBF liquid (**a**), Ringer’s solution (**b**), and distilled water (**c**), (*n*—number of repetitions, *n* = 3).

Incubation tests were performed to determine the hydrogen ion activity (pH) for the fluid in which the hydrogel matrices were incubated for a period of 20 days. The results obtained are shown in the graphs below (Figure 2).

**Figure 3:**
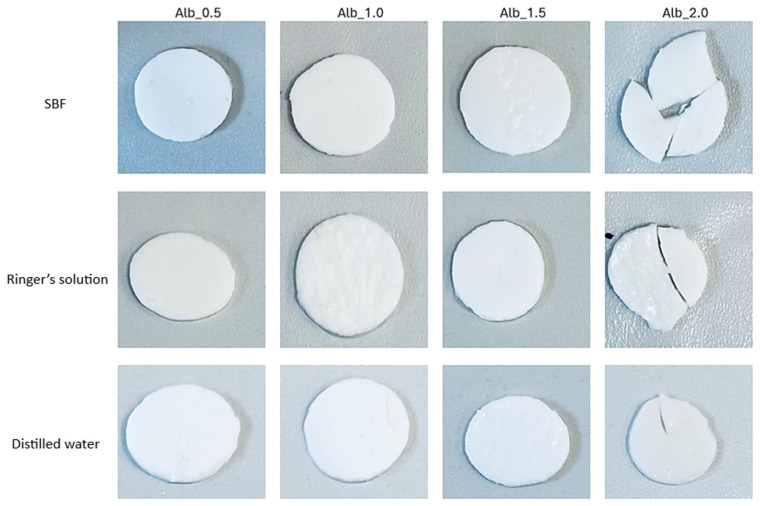
Samples images of multicomponent material after incubation analysis.

Monitoring pH changes during hydrogel incubation is essential for understanding the material’s interactions with its surroundings. Variations in pH can signal the release of substances, ongoing chemical reactions, or hydrolysis, which are crucial both for biomedical applications and for the controlled delivery of active compounds or other advanced hydrogel functionalities. A key focus is identifying hydrogel degradation, as this process can cause rapid and significant pH shifts. Consequently, this analysis was conducted to assess how hydrogels interact with fluids that mimic human physiological conditions. Temperature was also a critical factor, with samples incubated at 37 °C. The images below display hydrogel samples incubated in three selected fluids (Figure 3).

The results of the incubation study indicate that the hydrogel materials did not undergo degradation. The images above demonstrate that the samples maintained their shape and structure throughout incubation. However, samples containing 2.0 mL of protein carrier suspension exhibited cracks and structural damage in the polymer matrix, leading to their exclusion from further microscopic analysis. No abrupt or substantial pH fluctuations were observed. The SBF solution displayed the highest stability, functioning as a buffer that stabilizes pH levels even with the addition of small amounts of strong acids or bases. Minor pH changes (about one unit) were noted for samples in Ringer’s solution and distilled water, possibly indicating the release of active substances from the hydrogel matrix. These changes were minimal, and the samples preserved their structural integrity, confirming the absence of degradation.

### 2.3. Microscope Observations and Roughness Profile

Surface observations of the synthesized hydrogel materials were conducted using a digital microscope to allow visualization and comparison. Three samples with differing albumin content were selected for analysis. Additionally, these three samples were examined microscopically following the incubation test, with the exception of the sample containing 2 mL of protein suspension, which was excluded due to partial degradation. Table 2 presents the microscopic images obtained. Analysis of these images reveals that both the inclusion of plant extracts and variations in protein bead suspension content influenced the surface structure of the materials.

Next, observations were made using a scanning electron microscope. SEM images for samples observed at ×500 and ×1000 magnification are presented in Table 3.

Microscopic analysis revealed distinct variations in surface morphology across samples, influenced by the concentration of protein spheres. SEM images demonstrate that the sample with the lowest concentration of protein spheres exhibits a highly uniform and homogeneous structure, with a smooth, defect-free surface, indicating successful integration of protein spheres into the polymer matrix at lower levels. As the protein sphere concentration increases, the surface structure appears progressively more heterogeneous. Visible irregularities and pores emerge, likely due to challenges in achieving an even distribution of a higher number of spheres within the polymer matrix. This may lead to aggregation of protein spheres, forming clusters that disrupt the hydrogel’s uniformity. The observed inhomogeneity also reflects the polymer matrix’s limited capacity to crosslink uniformly around an increased sphere load, resulting in structural defects that could influence the material properties.

**Figure 4:**
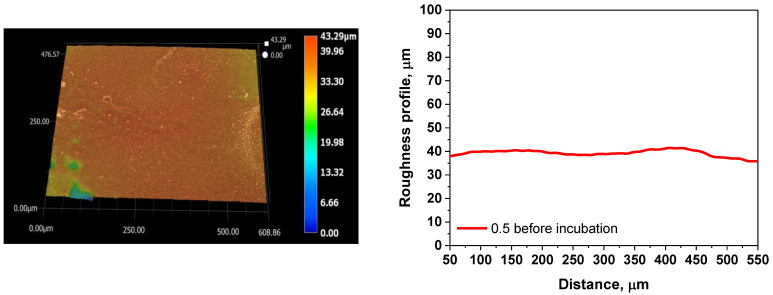

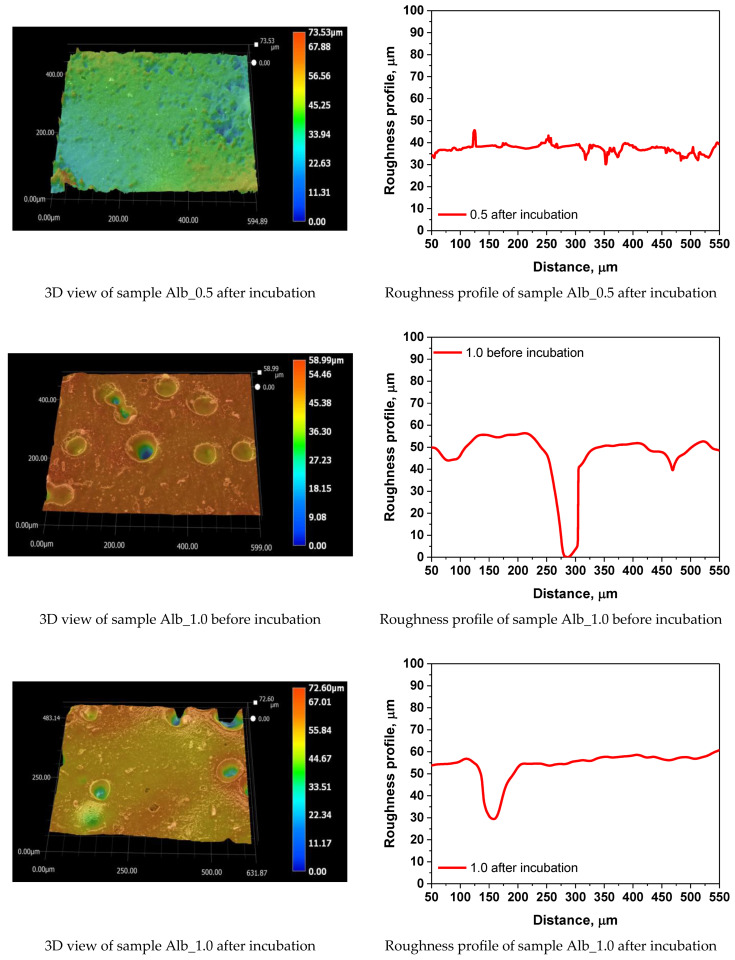

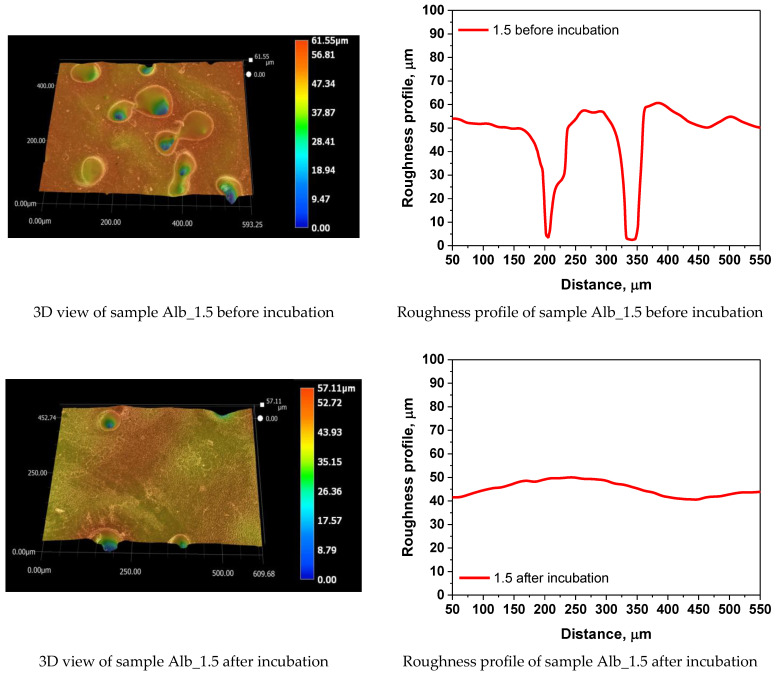
3D view and roughness profile of hydrogel samples before and after incubation.

The roughness parameters, along with the surface profile of the tested materials, are presented below in Table 4 and Figure 4.

The surface characteristics of polymeric materials play a crucial role in cell adhesion and proliferation, influencing the regenerative potential of damaged tissues. Surfaces with specific structural features, including appropriate roughness and corrugation, can serve as an ideal substrate for cellular attachment, whereas surfaces that are too rough or too smooth may impede cell adhesion and growth. Excessive surface roughness can make it challenging for cells to navigate across high elevations, while overly smooth surfaces can hinder adhesion at wound sites. Thus, an intermediate roughness level offers an optimal substrate for cell attachment and growth. For the tested materials, surface roughness, represented by the Ra parameter, ranged from 2.01 to 6.73 μm in samples before incubation and from 1.62 to 5.94 μm in samples post-incubation. Results indicate a reduction in Ra values after incubation, suggesting surface smoothing related to incubation processes. Due to their hydrophilic nature, hydrogels can absorb water, leading to swelling and smoothing of surface microstructures, which in turn reduces roughness. Furthermore, if the hydrogel matrix contains soluble substances, their release during incubation can impact the surface structure. Partial hydrolytic processes may also influence the polymer network’s structure, leading to rearrangement of polymer chains and subsequent surface smoothing. The results highlight the impact of the hydrogel composition on surface morphology, with variations in roughness observed across samples. The highest roughness was noted in the sample with the greatest protein suspension content, suggesting that increased albumin levels correlate with higher surface roughness. Both the incubation process and hydrogel composition were shown to affect surface properties, with no notable irregularities, clusters, or defects detected, indicating that the photopolymerization reaction proceeded as intended, yielding materials with a relatively uniform structure.

### 2.4. Infrared Spectroscopy with Fourier Transformation

**Figure 5:**
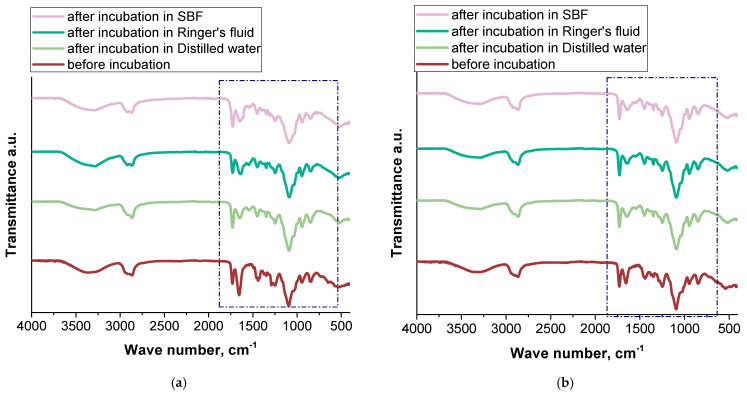

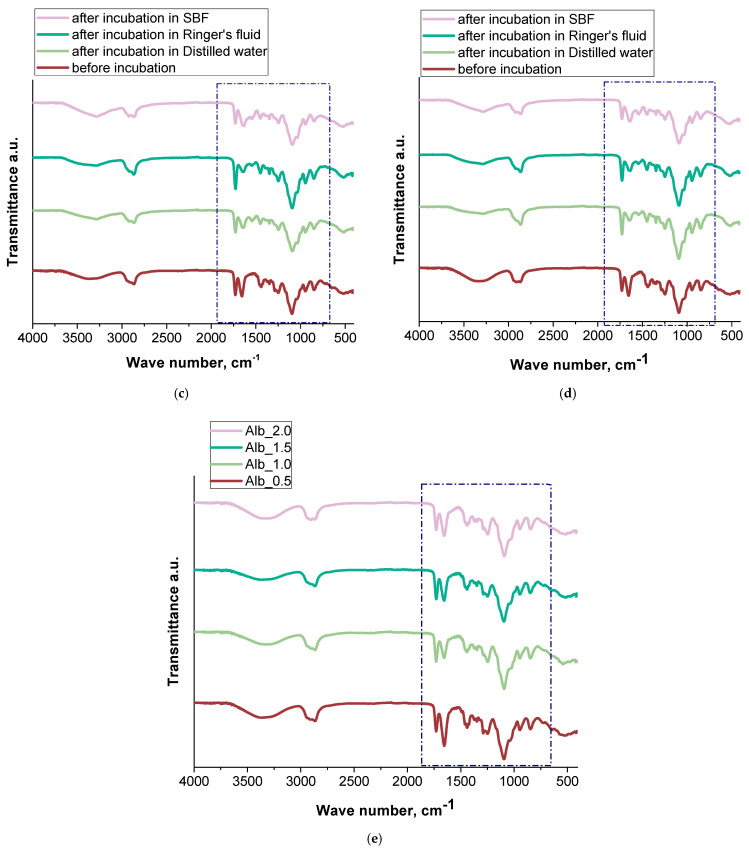
FT-IR spectra of hydrogel materials modified with albumin carries Alb_0.5 (**a**), Alb_1.0 (**b**), Alb_1.5 (**c**), Alb_2.0 (**d**), samples before incubation (**e**).

The incubation study was complemented by FT-IR spectroscopic analysis. The study made it possible to determine the effect of simulation fluids on the chemical structure of the tested hydrogels. The results are presented in Figure 5.

The FT-IR analysis identified key absorption bands corresponding to the functional groups present in the hydrogel matrix. A broad band in the range of 3500–3000 cm⁻^1^ was observed, attributed to the stretching vibrations of hydroxyl groups in polyvinyl alcohol (PVA) and -NH amine groups of albumin. The absorption band around 1700–1650 cm⁻^1^ was associated with the C=O carbonyl group of polyvinylpyrrolidone (PVP), while the band near 1550 cm⁻^1^ was characteristic of the -NH₂ amino group from albumin. These findings align with results from previous studies on hydrogels based on PVA and PVP, which demonstrated similar spectral profiles and stable polymeric networks [33,34,35]. No significant changes in these characteristic absorption bands were observed following incubation in simulated body fluids, indicating that no major structural degradation of the hydrogel materials occurred at a molecular level. However, the sample containing 2.0% albumin exhibited physical disintegration during the incubation process, despite the lack of distinct degradation signs in the FT-IR spectra. This suggests that while the chemical structure remained intact, the mechanical integrity or cohesive interactions within the hydrogel network may have been compromised. The higher albumin concentration could have influenced crosslinking density or water absorption properties, leading to swelling and eventual physical breakdown. These findings underscore the importance of optimizing the composition and structural properties of such hydrogel systems to ensure their stability and functionality in biomedical applications. The observed behavior provides valuable insights for future development, emphasizing the need to carefully balance the material’s mechanical and chemical properties to achieve reliable performance.

### 2.5. Wettability and Surface Energy Results

Next, the surface wettability results for the obtained hydrogel materials are presented in Table 5 and Table 6.

Water wetting angles in the range of 51–59° indicate a balance between the hydrophobic and hydrophilic properties of hydrogel surfaces. This range of wettability suggests that these materials can effectively adhere to moist wound surfaces, which is crucial for dressings in direct contact with tissue. As previous studies have shown, adequate wettability promotes even distribution of fluids on the wound surface, which can contribute to optimal drug release and the creation of the right microenvironment for the healing process [36,37,38].

Subsequently, diiodomethane wetting angle values in the range of 35–37° indicate good wettability by more non-polar liquids, suggesting compatibility with a variety of non-polar biomolecules and therapeutic substances (as do a significant proportion of drugs). This is particularly important for protein carriers containing anticancer drugs, as it may support stability and distribution of the drug in the wound environment [36,39,40].

The biocompatibility of hydrogels is a key factor in their medical applications. Appropriate wetting angles promote biological compatibility, minimizing the risk of irritation and inflammatory reactions. Materials that adhere well to moist surfaces, yet are not too hydrophobic, can promote natural healing processes [41]. In addition, controlled wettability can also affect drug release kinetics. Hydrogels can release protein carriers containing anticancer drugs in a continuous and controlled manner, which is beneficial for anticancer therapy. In addition, materials with adequate wettability can form a protective barrier against pathogens while allowing gas exchange and exudate removal, which is key to protecting the wound and promoting wound regeneration [42,43]. The uniformity of the wettability results suggests the stability and reproducibility of the surface properties of hydrogels, which is important for their predictable performance in practical medical applications. These materials, characterized by moderate wettability, can provide suitable conditions for wound healing, controlled drug release, and protection against infection. All this makes them promising candidates for biomedical applications, especially in the context of anticancer drug-releasing dressings.

Surface free energy is a key indicator of the adhesive properties of materials. The SFE values calculated for the tested samples indicate that the dispersion component ($\gamma_{s}^{d}$) dominates over the polar component ($\gamma_{s}^{p}$). This means that the tested materials have a higher capacity for dispersive interactions and a higher affinity for non-polar substances. The Owens–Wendt model, which assumes the division of energy into polar and dispersive components, allows a more accurate determination of the effect of different interfacial interactions on wettability and adhesion [44]. The $\gamma_{s}$ values for the tested materials range from 49.0 to 57.7 mJ/m^2^, with the highest value recorded for material Alb_1.5 (57.7 ± 2.1 mJ/m^2^), indicating its exceptionally good adhesive properties. The SEP value is dominated by the dispersion component, which may indicate a higher capacity for dispersion interactions and a higher affinity for non-polar substances. Dispersive interactions can promote the controlled release of drugs from the hydrogel. As a result, non-polar substances, such as some anticancer drugs, can be evenly distributed and gradually released, increasing the effectiveness of therapy. In terms of biocompatibility, materials with a predominant dispersion component may exhibit better biological compatibility. For example, a lower affinity for strong electrostatic interactions minimizes the risk of inflammatory reactions and irritation at the dressing application site. As a result, hydrogels can create a more friendly microenvironment for cells and tissues, promoting regenerative processes and wound healing [45].

In contrast, other researchers emphasize that effective binding of hydrogels to non-polar parts of biomolecules can increase the efficiency of drug delivery to target cells. In the case of protein carriers containing anticancer drugs, such hydrogel properties can support the stability and transport efficiency of these drugs. Moreover, in tissue engineering, materials with a predominant dispersion component can promote cell adhesion and intercellular matrix formation [46,47,48]. Hoffman et al. indicate that selected adhesive properties of hydrogels can lead to better tissue formation and faster wound healing. In addition, compatibility with a variety of substances makes it possible to integrate hydrogels with other biomedical materials, which opens up new possibilities in the design of advanced therapeutic systems [49].

### 2.6. Albumin Release Study

The aim of this study was to assess the release dynamics of albumin from two hydrogel systems with varying compositions, providing insights into their potential for controlled drug delivery applications. The release profiles obtained from these systems are presented below.

**Figure 6:**
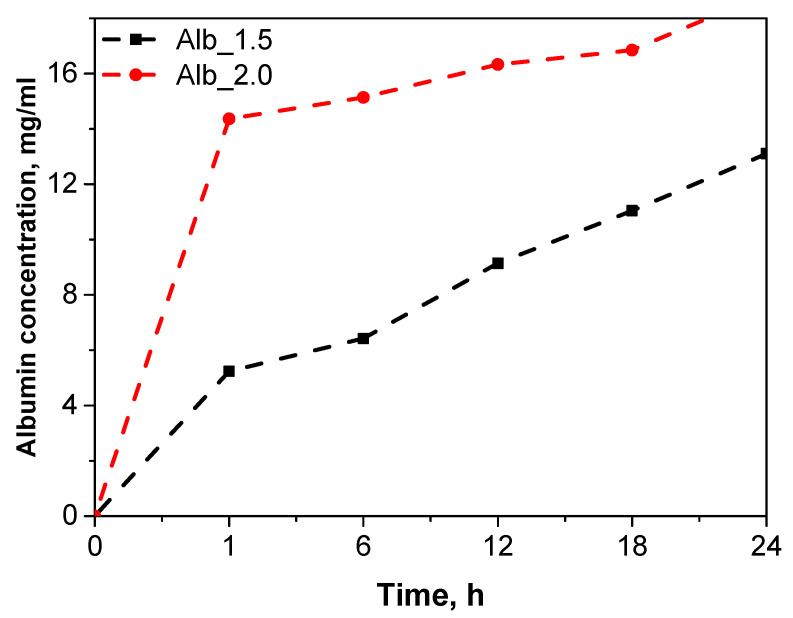
Albumin release profile from hydrogel systems.

The release profiles of albumin from hydrogels containing 1.5 mL and 2.0 mL of albumin suspension show significant differences, reflecting variations in structural stability and release dynamics (Figure 6). For the hydrogel with 1.5 mL of albumin, a gradual and stable increase in the concentration of released albumin was observed over time. At the start of the study, after 1 h, the released albumin concentration was 5.231 mg/mL, reaching 13.103 mg/mL at 24 h. This release profile indicates the preservation of the structural integrity of the hydrogel, enabling controlled diffusion of albumin from the polymer network.

In contrast, the hydrogel containing 2.0 mL of albumin exhibited a significantly higher initial burst of albumin release, reaching 14.363 mg/mL at 1 h and 18.897 mg/mL at 24 h. This sudden burst effect suggests issues with the structural stability of the hydrogel. As shown in previous studies, this hydrogel underwent partial disintegration during the tests, likely due to the higher albumin content, which may have affected the crosslinking density and water absorption capacity of the hydrogel. The weakened structural integrity could have contributed to the rapid release of albumin at the beginning of the study. The albumin release profiles from the tested hydrogels revealed significant differences depending on the albumin content in the hydrogel matrix. In the sample containing 1.5 mL of albumin, a stable and gradual release was observed, indicating the preservation of the hydrogel’s structural integrity. In contrast, the sample with 2.0 mL of albumin exhibited a rapid initial release of albumin (a “burst effect”) at the beginning of the study, which could be attributed to the weakened hydrogel structure. A similar effect was described in the study by Varnier et al., where the higher porosity of polysaccharide hydrogels promoted faster albumin release due to limited interactions between the protein and the polymeric network [50].

On the other hand, the study by Chen et al. demonstrated that controlled and more stable albumin release could be achieved through the use of hybrid hydrogel-nanocapsule systems, which enhance protein retention and extend the release duration [51].

## 3. Materials and Methods

### 3.1. Materials

Poly(vinyl alcohol) (PVA, crystalline powder, 87–89% hydrolyzed, Mw 13,000–23,000), polyvinylpyrrolidone (PVP, powder, average mol wt. 10,000), diacrylate poly(ethylene glycol) (crosslinking agent, PEGDA, average molecular weight Mn = 700 g/mol), and 2-hydroxy-2-methylpropiophenone (photoinitiator, 97%, d = 1.077 g/mL), potassium phosphate, and tris(hydroxymethyl)aminomethane (ACS reagent, 99.8%), cisplatin (solid, ≥98% (HPLC)) were applied during the preparation of composite materials. All mentioned reagents were purchased from Sigma Aldrich (Saint Louis, MO, USA). In turn, albumin (albumin egg powder) and hydrochloric acid (35–38%, d = 1.190 g/mL) were purchased from Avantor Performance Materials Poland S.A. (Gliwice, Poland). The plant products were purchased from Kawon (Gostyn, Poland). Chamomile was characterized in detail in [52,53,54], and common nettle in [55,56,57].

### 3.2. Synthesis of Hydrogel Materials

**Figure 7:**
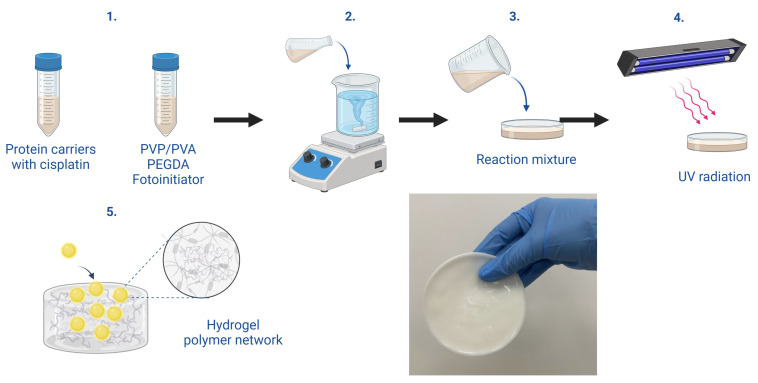
Scheme for synthesis of polymeric systems containing protein carriers with cytostatic drug.

The nettle and chamomile extracts were prepared following previously described methods. In both cases, the plant materials were extracted in heated or boiling water, filtered, and concentrated. For nettle, the preparation process has been detailed in [55], with its comprehensive characterization presented in [55,56,57]. For chamomile, the preparation method is described in [53], and its detailed characterization can be found in [52,53,54]. Next, a protein-desiccation technique was applied to produce a suspension of protein spheres. The synthesis process is comprehensively detailed in [58]. This suspension comprised albumin—a plasma protein with vital transport and regulatory roles—and cisplatin, a chemotherapeutic agent. The solution was initially dissolved in a Tris-HCl buffer at pH 7.5. Tripotassium phosphate (K_3_PO_4_) was then introduced as a desalting agent, which decreased albumin solubility and promoted its precipitation. Centrifugation was employed to separate the precipitated albumin, which was subsequently filtered and re-suspended in a PBS phosphate buffer, resulting in protein particles containing cisplatin. The quantity of cisplatin used during synthesis was calibrated to achieve a final concentration of 2 mg/mL within the protein suspension. The hydrogel synthesis involved preparing polyvinylpyrrolidone (PVP) and polyvinyl alcohol (PVA) solutions at specific concentrations, achieved through stirring with a magnetic stirrer. Specifically, a 10% PVA solution and a 15% PVP solution were used, maintaining a solution ratio and crosslinker combination of PVA:PVP as 1:1:0.36. The protein sphere suspension, poly(ethylene glycol) diacrylate (PEGDA 700) as a crosslinking agent, and a photoinitiator were then added to the reaction vessels in predetermined proportions. Nettle and chamomile extracts were also incorporated into each mixture. Nettle offers anti-inflammatory and antioxidant effects, whereas chamomile provides antibacterial and calming properties. After thorough mixing, the blend was poured into Petri dishes and subjected to UV polymerization for 2 min, using an EMITA VP-60 lamp (Famed, Łódź, Poland) (180 W, λ = 320 nm). The photopolymerization process initiates when the photoinitiator absorbs UV energy, decomposing into reactive radicals that instigate polymerization by reacting with the double bonds in PEGDA molecules. In the propagation phase, radicals continue to interact with monomers, forming extended polymer chains. PEGDA crosslinks the PVP and PVA chains, resulting in a three-dimensional polymeric network. This hydrogel structure demonstrates high water absorption and elasticity, attributable to the polymers’ intrinsic properties and the crosslinked network. Further modification of the hydrogel was achieved by embedding protein carriers loaded with cisplatin into the pre-polymerized mixture. This ensured the uniform distribution of protein particles within the hydrogel matrix, facilitating their stable integration into the structure. The procedural steps for synthesizing these hydrogel materials are illustrated in Figure 7.

After the polymerization process was completed, the finished hydrogels were taken out of the Petri dishes, and the discs with a diameter of 1 cm were cut out and left to dry. The composition of each hydrogel is shown in Table 7 (quantities were presented for the polymer base mixture of 5 mL of PVP and 5 mL of PVA). All matrices were labeled with the corresponding sample numbers for further analysis. The sample number indicates the content of the protein suspension in the hydrogel.

All matrices have been marked with the appropriate sample numbers and composition of the components, which will allow for further analysis and comparison.

### 3.3. Sorption Capacity Analysis

The objective of this analysis was to comprehensively assess the sorption properties of hydrogel matrices with regard to their swelling behavior. This investigation aimed to explore the potential of hydrogel dressings for efficient absorption of therapeutic fluids. Pre-prepared and dried hydrogel disks were used as the test samples. Initially, each sample’s weight was precisely measured using an analytical balance before placing it in three incubation fluids: simulated body fluid (SBF), Ringer’s solution, and distilled water, which emulate human physiological conditions.

The samples were then incubated in these fluids for designated durations of 1, 6, and 12 h. After each incubation period, any excess fluid was carefully removed from the surface of the disks, and the samples were weighed again with a Radwag analytical balance to ensure precision. To determine the sorption capacity of the hydrogel matrices, the swelling coefficient values obtained from repeated trials were averaged, providing a reliable representative measurement. The swelling factor, indicating sorption capacity, was derived by calculating the sorption coefficient, α.

(1) $$\alpha =\frac{\left(m_{t}-m_{0}\right)}{m_{0}}$$

where:

α—swelling ratio, g/g;

m_t_—mass of swollen sample after time “t”, g;

m_0_—mass of dry sample (before the study), g.

The chemical composition of the SBF fluid and Ringer’s solution is shown in Table 8 and Table 9.

### 3.4. Incubation Studies in Simulated Body Fluids

The incubation study aimed to demonstrate the interaction between the hydrogel matrix and solutions that simulate human physiological fluids. Monitoring pH changes can reveal the leaching of uncross-linked components or sample degradation in these fluids. For the experiment, hydrogel samples, each with a 1 cm diameter, were aseptically placed in containers with 50 mL of each incubation solution, specifically SBF, Ringer’s solution, and distilled water. These solutions were selected due to their distinct chemical properties: SBF, a simulated body fluid, facilitates the evaluation of hydrogel behavior in a physiologically similar environment; Ringer’s solution, with an ionic composition akin to bodily fluids, helps assess the hydrogel’s response to biological conditions critical for material design. Distilled water served as a control in the analysis. The samples were incubated at 37 °C, and pH levels were recorded using a CX-701 ELMETRON multifunctional device (Elmetron, Zabrze, Poland) over a 20-day period.

### 3.5. Microscope Observations and Roughness Profile

To analyze the surface and structure of the synthesized polymer matrices, observations were made using Keyence’s VKX-700 advanced digital microscope, capable of accurately producing images in 4K resolution (Keyence International, Mechelen, Belgium). This state-of-the-art microscope is equipped with a CEO REMAX optical engine and a 4K CMOS image sensor for high accuracy and magnification. Microscopic studies were carried out at ×500 magnification. The main purpose of these observations was to analyze the morphology of hydrogel surfaces resulting from modifying the amount of protein sphere suspension and introducing additional modifiers into their matrices, as well as to determine roughness profiles.

SEM analysis was performed for hydrogel samples that had been dried and sputtered with a layer of nanogold. The observations were made using a scanning electron microscope JOEL IT200 (JEOL Ltd., Peabody, MA, USA).

### 3.6. Infrared Spectroscopy with Fourier Transformation

FT-IR spectroscopy was utilized to identify distinctive functional hydrogels, both unmodified and those infused with different protein concentrations. The analysis was conducted using the Nicolet iS5 Thermo Scientific spectrometer from Thermo Fisher Scientific (Loughborough, UK). Spectra were captured across the 4000–500 cm^−1^ range with a resolution of 4.0 cm^−1^ at room temperature.

### 3.7. Wetting Angle Measurement Methodology

The wetting angle (θ) was determined using the sessile drop method. An optical goniometer (Advex Instrument, Brno, Czech Republic) with SeeSystem software (http://www.advex-instruments.cz/) was employed to record and analyze the image of the droplet applied to the material surface. Ultra-pure distilled water (Biomus, Lublin, Poland) and diiodomethane (Sigma Aldrich, Poznań, Poland) were employed as the measuring fluids. These two liquids are commonly employed in the determination of the surface free energy of solids, for instance, through the Owens–Wendt method. They are selected in such a way that one of them is characterized by the highest possible value of the polar component (water—$\gamma_{l}^{d}$ = 21.8 mJ/m^2^ and $\gamma_{l}^{p}\;$= 51.0 mJ/m^2^) and the other is a dispersive liquid (diiodomethane—$\gamma_{l}^{d}$ = 50.8 mJ/m^2^ and $\gamma_{l}^{p}\;$= 0) [59]. For each material and measuring liquid, 10 measurements were taken by applying a 0.5 μL drop to the surface of the material using a micropipette. The test was conducted at a temperature of 22 °C and a humidity of 45%. The measured wetting angle values were employed to calculate the surface free energy (SFE, γs) using the Owens–Wendt method. This method is predicated on the assumption that the surface free energy of a solid is the sum of the polar component ($\gamma_{s}^{p}$) and the dispersive component ($\gamma_{s}^{d}$). These components are calculated using Equation (2), whereby a system of two equations of the same form is created, with the constant coefficients differing for a given measurement fluid.

(2) $$\frac{1}{2}\left(1+cos\theta \right)\gamma_{L}=\sqrt{\gamma_{S}^{d}\gamma_{L}^{d}}+\sqrt{\gamma_{S}^{p}\gamma_{L}^{p}}$$

where:

$\gamma_{S}^{d}$—dispersive component of SFE of tested material,

$\gamma_{S}^{p}$—polar component of SFE of tested material,

$\gamma_{L}$—surface free energy of measurement liquid,

$\gamma_{L}^{d}$—dispersive component of SFE of a liquid,

$\gamma_{L}^{p}$—polar component of SFE of a liquid,

θ—measured contact angle.

### 3.8. Albumin Release Study

The albumin release study from hydrogel samples was conducted in simulated body fluid (SBF) to mimic physiological conditions. Hydrogel samples were placed in 50 mL of pre-prepared SBF at 37 °C and incubated in a shaking incubator to ensure uniform experimental conditions.

At predefined time points (0, 1, 6, 12, 18, and 24 h), 2 mL of the fluid was withdrawn and replaced with an equal volume of fresh SBF to maintain stable dynamic equilibrium conditions. The collected samples were analyzed using UV-Vis spectrophotometry at a wavelength of 280 nm, corresponding to the absorption maximum of albumin. The concentration of released albumin was determined based on a calibration curve prepared using standard albumin solutions. Spectrophotometric determination of proteins was performed according to the methodology for protein identification set out in previous work such as [60,61].

## 4. Conclusions

The objective of this study was to develop hydrogel materials suitable for use as dressings or coatings. The photopolymerization method employed enabled the creation of hydrogels with varying compositions, particularly in terms of protein sphere content, while maintaining a consistent level of natural additives—namely, common nettle and chamomile. The protein spheres were prepared through a desalting process. Physicochemical analyses were conducted to assess the impact of this modification on selected material properties. A notable increase in sorption capacity was observed during the first hour of testing, followed by a gradual stabilization of swelling coefficients over time. The findings confirmed that hydrogel sorption capacity was highest in distilled water compared to Ringer’s solution and SBF. Incubation studies, along with pH measurements, ruled out material degradation and supported their compatibility with fluids that mimic physiological conditions. Microscopic analysis, along with surface roughness measurements, demonstrated a clear correlation between composition modifications and surface structure changes. After incubation, all materials exhibited significantly lower surface roughness than prior to incubation. The composition of the polymer matrix was also shown to influence surface morphology, with the sample containing the highest albumin content displaying the greatest roughness. Results indicated that surface roughness increased with higher protein sphere content, although no agglomerates were detected, suggesting system homogeneity. In conclusion, the study successfully yielded hydrogels capable of fluid absorption with a textured surface, presenting potential applications as dressing materials, particularly for the controlled delivery of cytostatic drugs.

While the findings of this study are promising, further research is needed to expand the scope of these materials’ applications. In particular, additional investigations, including in vivo studies, could provide valuable insights into their performance and compatibility in biological environments. These efforts would contribute to validating their potential for practical biomedical use and optimizing their properties for specific clinical needs.

## Figures and Tables

**Table 1:** Statistical analysis of obtained data based on the two-way analysis of variance (ANOVA) (for sorption ability after 12 h).

Independent Variable	Sum of Squares	Mean Square	f-Value	*p*-Value
Type of incubation fluid	1.69839	0.8492	6420.74578	1.23 × 10^−4^
Composition of sample	4.63471	1.5449	11,680.97135	3.93 × 10^−9^
Interaction	2.23355	0.37226	2814.63533	0.048

At the 0.05 level, the population means of “type of incubation fluid” is significantly different. At the 0.05 level, the population means of “composition of sample” is significantly different. At the 0.05 level, the interaction between both factors is significant.

**Table 2:** Digital microscope images before and after incubation and samples containing plant extract.

Sample Number	Before Incubation	After Incubation
Alb_0.5	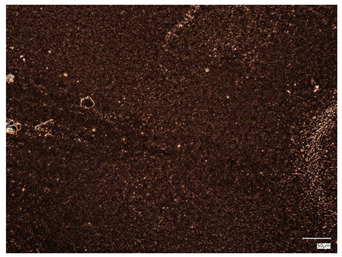	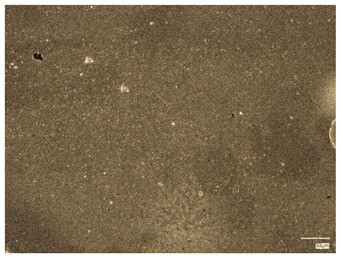
Alb_1.0	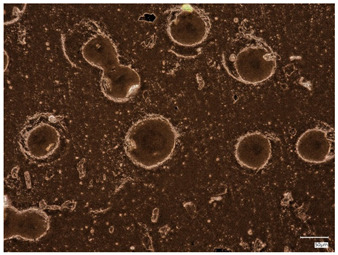	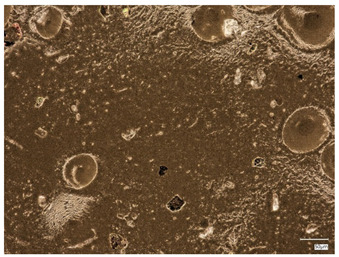
Alb_1.5	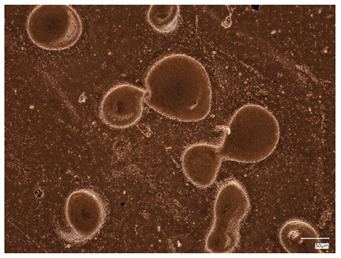	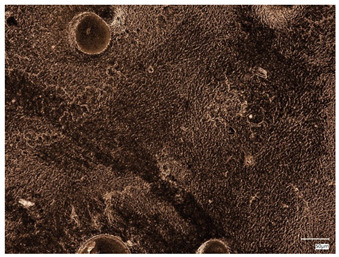

**Table 3:** SEM images of the obtained hydrogel materials.

**Sample Name**	**×500**	**×1000**
Alb_0.5	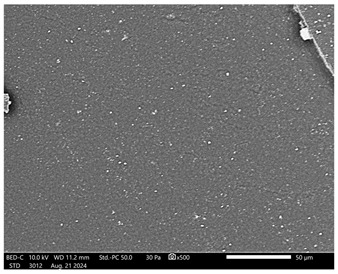	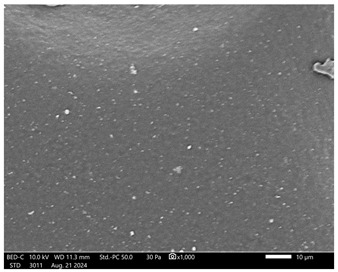
Alb_1.0	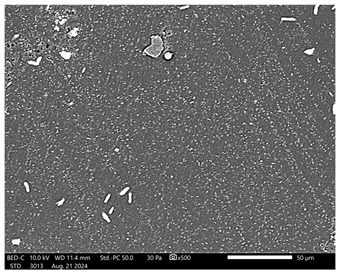	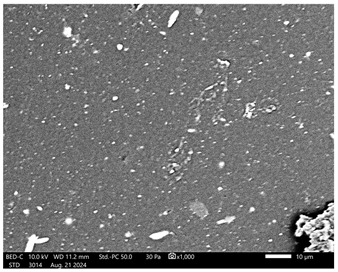
Alb_1.5	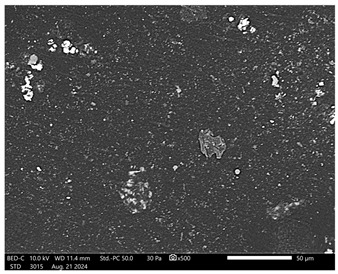	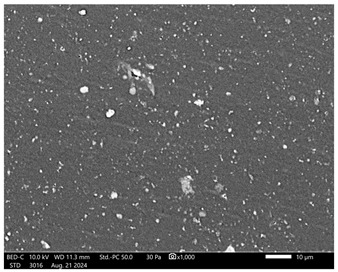
Alb_2.0	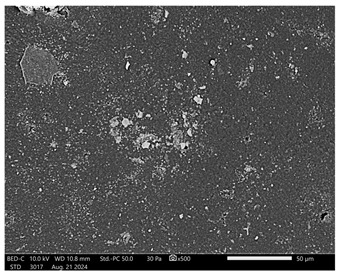	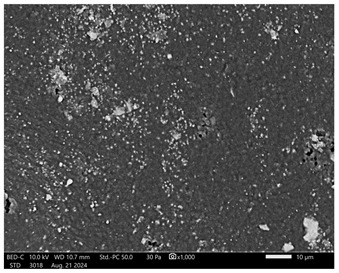

**Table 4:** Roughness parameters of hydrogel materials.

Sample Number	Ra [μm] Before Incubation	Ra [μm] After Incubation
Alb_0.5	2.01	1.62
Alb_1.0	4.75	2.68
Alb_1.5	6.73	5.94

**Table 5:** Exemplary liquid drops’ shape of materials.

Material	Water	Diiodomethane
Alb_0.5	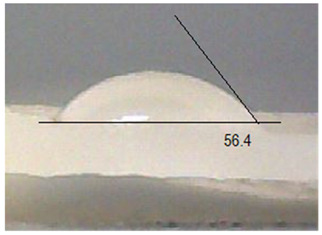	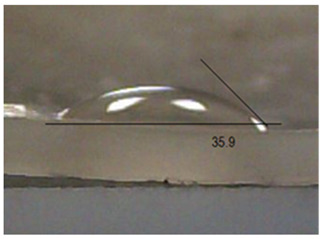
Alb_1.0	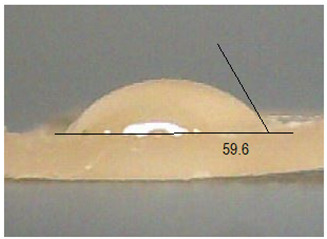	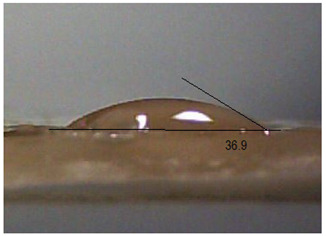
Alb_1.5	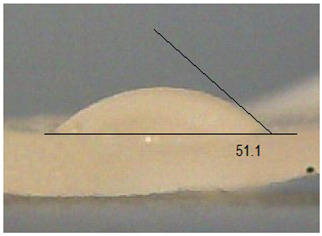	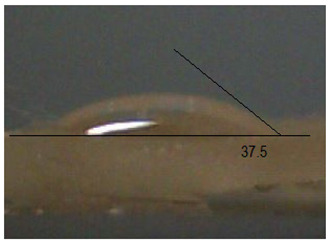
Alb_2.0	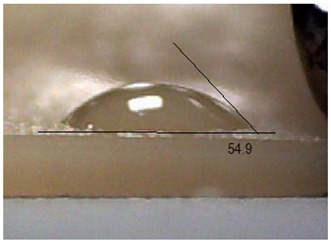	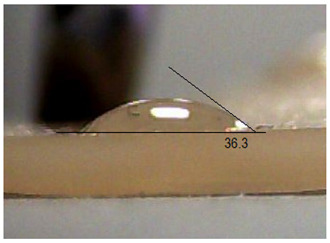

**Table 6:** Comparison of the contact angle, SFE, and its components (mean value ± standard deviation).

	Contact Angle [°]		SFE and Its Components [mJ/m^2^]		
**Sample Number**	**Water**	**Diiodomethane**	$\gamma_{s}$	$\gamma_{s}^{d}$	$\gamma_{s}^{p}$
Alb_0.5	56.4 ± 2.2	35.9 ± 3.8	55.3 ± 1.6	41.4 ± 1.7	13.9 ± 1.4
Alb_1.0	59.6 ± 1.5	36.9 ± 6.4	53.1 ± 2.4	40.8 ± 3.0	12.3 ± 1.0
Alb_1.5	51.1 ± 3.2	37.5 ± 2.9	57.7 ± 2.1	40.6 ± 1.5	17.1 ± 1.9
Alb_2.0	54.9 ± 2.2	36.3 ± 3.6	55.9 ± 2.1	41.2 ± 1.7	14.7 ± 1.0

**Table 7:** Composition of hydrogel materials.

Sample Name	Protein Spheres Suspension [mL]	Common Nettle [mL]	Common Chamomile [mL]	PEGDA 700 [mL]	Photoinitiator [µL]
Alb_0.5	0.5	0.5	0.5	1.8	70
Alb_1.0	1.0				
Alb_1.5	1.5				
Alb_2.0	2.0				

**Table 8:** Composition of Ringer’s solution.

	Component	Amount [g/L]
1	NaCl	8.600
2	KCl	0.300
3	CaCl_2_·H_2_O	0.480

**Table 9:** Composition of SBF.

	Component	Amount [g/L]
1	NaCl	8.035
2	NaHCO_3_	0.355
3	KCl	0.225
4	K_2_HPO_4_·3H_2_O	0.231
5	MgCl_2_·6H_2_O	0.311
6	1M HCl	39 ml
7	CaCl_2_	0.292
8	Na_2_SO_4_	0.072
9	Tris	6.118

## Data Availability

The data that support the findings of this study are contained within the article.
